# Self-(in)compatibility inheritance and allele-specific marker development in yellow mustard (*Sinapis alba*)

**DOI:** 10.1007/s11032-013-9943-8

**Published:** 2013-09-22

**Authors:** Fangqin Zeng, Bifang Cheng

**Affiliations:** Agriculture and Agri-Food Canada, Saskatoon Research Centre, 107 Science Place, Saskatoon, SK S7N 0X2 Canada

**Keywords:** Yellow mustard (*Sinapis alba*), Self-(in)compatibility, Inheritance, *S*-locus allele-specific marker, Marker-assisted breeding

## Abstract

Yellow mustard (*Sinapis alba*) has a sporophytic self-incompatibility reproduction system. Genetically stable self-incompatible (SI) and self-compatible (SC) inbred lines have recently been developed in this crop. Understanding the *S* haplotype of different inbred lines and the inheritance of the self-(in)compatibility (SI/SC) trait is very important for breeding purposes. In this study, we used the *S*-locus gene-specific primers in *Brassica rapa* and *Brassica oleracea* to clone yellow mustard *S*-locus genes of SI lines Y514 and Y1130 and SC lines Y1499 and Y1501. The PCR amplification results and DNA sequences of the *S*-locus genes revealed that Y514 carried the class I *S* haplotype, while Y1130, Y1499, and Y1501 had the class II *S* haplotype. The results of our genetic studies indicated that self-incompatibility was dominant over self-compatibility and controlled by a one-gene locus in the two crosses of Y514 × Y1499 and Y1130 × Y1501. Of the five *S*-locus gene polymorphic primer pairs, Sal-SLGI and Sal-SRKI each generated one dominant marker for the SI phenotype of Y514; Sal-SLGII and Sal-SRKII produced dominant marker(s) for the SC phenotype of Y1501 and Y1499; Sal-SP11II generated one dominant marker for Y1130. These markers co-segregated with the SI/SC phenotype in the F_2_ populations of the two crosses. In addition, co-dominant markers were developed by mixing the two polymorphic primer pairs specific for each parent in the multiplex PCR, which allowed zygosity to be determined in the F_2_ populations. The SI/SC allele-specific markers have proven to be very useful for the selection of the desirable SC genotypes in our yellow mustard breeding program.

**Electronic supplementary material:**

The online version of this article (doi:10.1007/s11032-013-9943-8) contains supplementary material, which is available to authorized users.

## Introduction

The tribe Brassiceae comprises many self-incompatible (SI) species, such as *Brassica rapa*, *Brassica oleracea*, *Raphanus sativus*, and *Sinapis alba*. Many genetic and molecular studies have been carried out on the self-(in)compatibility (SI/SC) mechanism in *B. rapa* and *B. oleracea*. It is now well understood that this trait is sporophytically controlled by multiple alleles of a single locus, the *S* locus, in Brassiceae (Bateman 1955; Nasrallah and Nasrallah 1993). Three genes located in the *S* locus, namely, *S*-receptor kinase (SRK), a female determinant, *S*-locus protein 11/*S*-locus cysteine-rich protein (SP11/SCR), a male determinant, and *S*-locus glycoprotein (SLG), which can enhance the SI recognition process, have been characterized (Nasrallah et al. 1988; Schopfer et al. 1999; Stein et al. 1991; Suzuki et al. 1999; Watanabe et al. 2001). As meiotic recombination between the three genes of the *S* locus seldom occurs, a set of alleles of *SRK*, *SLG*, and *SP11*, termed as the *S* haplotype, is inherited by the progeny. About 30 and 50 *S* haplotypes have been identified in *B. rapa* and *B. oleracea*, respectively (Nou et al. 1993; Ockendon 2000). Different *S* haplotypes have been classified into two groups, class I and class II (Nasrallah et al. 1991). Class I *S* haplotypes are generally dominant over class II *S* haplotypes and prevalent in *B. oleracea* and *B. rapa* (Nasrallah and Nasrallah 1993; Sato et al. 2006). Molecular studies have revealed that the DNA sequences of *SLG* genes share a higher degree of similarity within the class I *S* haplotypes than those between class I and class II types (Nasrallah et al. 1991).

In *Brassica* species, the identification of different *S* haplotypes has been classically carried out by crossing lines with an unknown *S* haplotype to tester lines of a known *S* haplotype (Ruffio-Châble 1998). However, this method cannot be used to distinguish the *S* haplotypes of partial self-compatible (SC) lines since it is based on the pollen-tube growth and seed setting. Biochemical methods have been developed to identify different *S* haplotypes by analyzing the specific *S*-glycoproteins produced by the *S*-locus gene (Nasrallah et al. 1991; Ruffio-Châble et al. 1997). More recently, the PCR-restriction fragment length polymorphism (PCR-RFLP) technique has been successfully used as a molecular approach to identify *S* haplotypes in *B. rap*a (Nishio et al. 1996), *B. olerace*a (Brace et al. 1993; Nishio et al. 1997), and *R. sativus* (Sakamoto et al. 1998; Lim et al. 2002). In these studies, primer pairs specific for the *SLG* and *SRK* genes were used to amplify the DNA fragments of different *S* haplotypes. The resulting amplified DNA fragments exhibited different electrophoretic profiles after cleavage with restriction endonuclease(s), and these DNA fragments have proven to be useful markers for the identification of different *SLG* or *SRK* alleles.

Yellow mustard (*S. alba*) (genome: SS, 2*n* = 24) is grown as an important condiment crop for the spice trade throughout the world. Similar to *B. rapa* and *B. oleracea*, yellow mustard is an obligate outcrossing species due to its sporophytic self-incompatibility. However, there has been a lack of genetic and molecular studies on the SI/SC trait in this crop. Recently, different SI and SC inbred lines have been produced with the objective to develop high-yielding synthetic varieties in yellow mustard (Cheng et al. 2012). In this context, characterization of the *S* haplotypes of different inbred lines and an understanding of the genetics of SI/SC trait are essential for breeding programs. In addition, the development of allele-specific diagnostic markers for the SI/SC phenotype will greatly increase breeding efficiency. Therefore, the objectives of our study were: (1) to clone and sequence the *S*-locus genes and classify the *S* haplotypes of two SI and two SC inbred lines, (2) to study the inheritance of the SI/SC trait, (3) to develop *S*-locus gene allele-specific markers for marker-assisted breeding in yellow mustard.

## Materials and methods

### Plant materials

Two yellow mustard SI lines, Y514 and Y1130, and two SC lines, Y1499 and Y1501, were used in this study. Y514 is a doubled haploid line, and Y1130 is an inbred line developed from the breeding line W96-1-2 (Cheng et al. 2012; Javidfar and Cheng 2013). Y1499 and Y1501 are S5 inbred lines derived from the open-pollinated plants Y041-1 and Y020-11 of cv. Andante, respectively. Y514 and Y1130 were crossed as the female with Y1499 and Y1501, respectively, to produce F_1_ hybrid seeds. The hybridity of the F_1_ plants was confirmed using molecular markers specific for each parent. The main racemes of the hybrid F_1_ plants were bagged for self-pollination, while the branches were bud-pollinated to produce F_2_ seeds. All F_2_ plants were bagged for self-pollination.

### Measurement of the self-compatibility index

The self-compatibility index (SCI) (number of seeds/self-pollinated pod) of the parental, F_1_, and F_2_ plants was measured according to Cheng et al. (2012). At least ten plants of each of the four parental lines and ten F_1_ hybrid plants of each of the two crosses were measured to determine the SCI. The SI lines Y514 and Y1130 had an average SCI of 0.5 (range 0.0–1.1) and 0.05 (range 0.0–0.1), respectively. The SC lines Y1499 and Y1501 had an average SCI of 4.2 (range 3.0–6.0) and 4.8 (range 3.2–6.9), respectively; these are similar to the seed setting (5.4 seeds/pod) of the open-pollinated plants in yellow mustard (Olsson 1974). Segregation of the SI/SC trait was studied in the F_2_ populations of Y514 × Y1499 and Y1130 × Y1501. The F_2_ plants were classified into two groups according to the SCI: (1) plants with a low SCI (0.0–1.2) similar to that of the SI lines Y514 and Y1130, (2) plants with a high SCI (3.0–6.4) similar to that of the SC lines Y1499 and Y1501. The χ^2^ goodness-of-fit test was used to determine the SI/SC inheritance model. All plants were grown in the greenhouse at the Agriculture and Agri-Food Canada-Saskatoon Research Centre.

### Development of allele-specific markers for self-(in)compatibility

Twelve *S*-locus-specific primer pairs of *B. rapa* and *B. oleracea* (Table 1) were used to amplify the *S*-locus genes in yellow mustard. The primer pair PS5 + PS15 (No. 1) was designed based on the DNA sequences of *SLG* genes of class I *S* haplotypes *S*-*8* and *S*-*6* in *B. rapa* and *B. oleracea*, respectively (Nishio et al. 1996). The primer pair PK1 + PK4 (No. 2) was designed based on the *SRK* gene sequence of class I *S* haplotype *S*-*6* in *B. oleracea* and used to amplify the second to fifth exon of the *SRK* gene (Nishio et al. 1997). The primer pair PS3 + PS21 (No. 3) is specific for the *SLG* gene of the class II *S* haplotype *S*-*2* in *B. oleracea* (Nishio et al. 1996). The primer pair PK7-2II + PK8-2II (No. 4) is specific for the class II *SRK* gene *in B. rapa* (Fukai et al. 2003). The *SRK* and *SP11* gene sequences have been reported for the class II *S* haplotypes *S*-*29*, *S*-*44*, *S*-*40*, and *S*-*60* in *B. rapa* and *S*-*15* in *B. oleracea* (Fujimoto et al. 2006; Hatakeyama et al. 1998; Kakizaki et al. 2006; Shiba et al. 2002). The conserved regions of the *SRK* and *SP11* genes of the above-mentioned class II *S* haplotypes were analyzed using the CLUSTAL × program, respectively (Thompson et al. 1997). Based on the results, we designed seven primer pairs (No. 5–11) to amplify different exons and introns of the class II *SRK* gene and one primer pair (No. 12) to amplify the *SP11* gene in search of polymorphic markers in yellow mustard.

**Table 1:** Primers designed based on the sequences of the *SLG*, *SRK*, and *SP11* genes of different *S* haplotypes in *Brassica* species

No.	Primer	Sequence (5′–3′)	Length (bp)	Source	*S* haplotype	Species
1	PS5	ATGAAAGGCGTAAGAAAAACCTA	23	*SLG*-*8* (1–23)^a^	Class I	*B. rapa*
	PS15	CCGTGTTTTATTTTAAGAGAAAGAGCT	27	*SLG*-*6* (1,336–1,310)	Class I	*B. oleracea*
2	PK1	CTGCTGATCATGTTCTGCCTCTGG	24	Second exon of *SRK* gene	Class I	*B. oleracea*
	PK4	CAATCCCAAAATCCGAGATCT	21	Fifth exon of *SRK* gene	Class I	*B. oleracea*
3	PS3	ATGAAAGGGGTACAGAACAT	20	*SLG*-*2A* (1–20)^a^	Class II	*B. oleracea*
	PS21	CTCAAGTCCCACTGCTGCGG	20	*SLG*-*2A* (1,025–1,006)	Class II	*B. oleracea*
4	PK7-2II	ATGAAAAGGGTACAGAACATTTACCACC	28	*SRK* gene	Class II	*B. rapa*
	PK8-2II	CCAGTTCGGTCTCTCTTCTCACCCGAGG	28	*SRK* gene	Class II	*B. rapa*
5	SRKII-1L	CCACCATTCTTACACCTTCT	20	First exon of *SRK* gene	Class II	*B. rapa* and *B. oleracea*
	SRKII-1R	AGATCAGCAGCATTCAATCT	20	First exon of *SRK* gene	Class II	*B. rapa* and *B. oleracea*
6	SRKII-2L	TACGTCAGATTGAATGCTGCTG	22	First intron of *SRK* gene	Class II	*B. rapa* and *B. oleracea*
	SRKII-2R	GTAACACCACCTCGTTCATTAG	22	Second exon of *SRK* gene	Class II	*B. rapa* and *B. oleracea*
7	SRKII-3L	AGTTCTAATGAACGAGGTGG	20	Third exon of *SRK* gene	Class II	*B. rapa* and *B. oleracea*
	SRKII-3R	GAGGAATAATAGGAGATACG	20	Third intron of *SRK* gene	Class II	*B. rapa* and *B. oleracea*
8	SRKII-4L	GTATCTCCTATTATTCCTCA	20	Third intron of *SRK* gene	Class II	*B. rapa* and *B. oleracea*
	SRKII-4R	CACATGCGGTCATATTATTC	20	Third intron of *SRK* gene	Class II	*B. rapa* and *B. oleracea*
9	SRKII-5L	TAATATGACCGCATGTGCTG	20	Third intron of *SRK* gene	Class II	*B. rapa* and *B. oleracea*
	SRKII-5R	TTGATGGCCTGAGAATATCC	20	Third intron of *SRK* gene	Class II	*B. rapa* and *B. oleracea*
10	SRKII-6L	TGTCAGCTCAAGGTACCGAT	20	Third exon of *SRK* gene	Class II	*B. rapa* and *B. oleracea*
	SRKII-6R	CTGACTTCATCGAGAATGTC	20	Fourth exon of *SRK* gene	Class II	*B. rapa* and *B. oleracea*
11	SRKII-7L	TCCAGAATATGCGATGAACG	20	Fifth exon of *SRK* gene	Class II	*B. rapa* and *B. oleracea*
	SRKII-7R	TACCGAGCGTCAATGATCGA	20	Seventh exon of *SRK* gene	Class II	*B. rapa* and *B. oleracea*
12	SP11II-L	TTGCATAGAGTAACCGTCTC	20	*SP11* gene	Class II	*B. rapa* and *B. oleracea*
	SP11II-R	CCGTCGTATATTGCATAGAGTA	22	*SP11* gene	Class II	*B. rapa* and *B. oleracea*

*SRK S*-receptor kinase gene, *SCR* *S*-cysteine-rich protein gene, *SLG* *S*-locus glycoprotein gene, *SP11* *S*-locus protein 11 gene

^a^Number in parenthesis indicates the position of the nucleotide sequence

The *SLG*, *SRK*, and *SP11* gene fragments were cloned and sequenced from yellow mustard lines Y514, Y1130, Y1499, and Y1501 using the primer pairs in Table 1. Based on the *SLG*, *SRK*, and *SP11* gene sequences of the four lines, we designed five primer pairs, namely, Sal-SLGI, Sal-SRKI, Sal-SLGII, Sal-SRKII, and Sal-SP11II (Table 2), for the generation of SI/SC allele-specific markers using Primer3 software (http://redb.croplab.org/modules/redbtools/primer3.php). Co-segregation of the allele-specific markers with the SI and SC phenotypes was investigated in the F_2_ populations from two crosses: Y514 × Y1499 and Y1130 × Y1501. In addition, a multiplex PCR mixture containing polymorphic primer pairs for the two parents was used to generate co-dominant markers for zygosity determinations in segregating F_2_ populations.

**Table 2:** Polymorphic primer pairs designed based on the DNA sequences of *S*-locus genes in yellow mustard (*S. alba*)

Primer name	Nucleotide sequences	Annealing temp (°C)	Size (bp)	Amplified region	Marker type
Sal-SLGI	5′-ACTTCGTGATGCGAGACTCC-3′	68	626	(419–438)^a^	Dominant
	5′-CCGCGTCTTCCTCATACACC-3′			(1.026–1.045)^a^	
Sal-SRKI	5′-GATTATCTCGTGTCTGAATG-3′	58	640	Second intron of *SRK* gene	Dominant
	5′-GGTAATGTCGAATCTCTCCT-3′			Fifth exon of *SRK* gene	
Sal-SLGII	5′-GGGATTGCCTGAGTTTGTTC-3′	60	310	(631–650)^a^	Dominant
	5′-TGTCGCAATAAGCATAAGCC-3′			(925–944)^a^	
Sal-SRKII	5′-TACGTCAGATTGAATGCTGCTG-3′	60	1,000/1,200	First intron of *SRK* gene	Dominant
	5′-GTAACACCACCTCGTTCATTAG-3′			Second exon of *SRK* gene	
Sal-SP11II	5′-TTGCATAGAGTAACCGTCTC-3′	60	420	*SP11* gene	Dominant
	5′-CCGTCGTATATTGCATAGAGTA-3′			*SP11* gene	

^a^Number in parenthesis indicates the position of the nucleotide sequence

### DNA extraction, PCR, and DNA sequencing

Genomic DNA was extracted from the young expanding leaves of the parental lines (Y514, Y1130, Y1499, Y1501) and F_1_ and F_2_ plants using a modified sodium dodecyl sulfate method (Somers et al. 1998). The PCR reaction mixture (20 μl) contained 1× PCR buffer, 1.5 mM MgCl_2_, 200 μM of each dNTP, 0.1 μM of each forward and reverse primer, 1 U of* Taq* polymerase (NEB, Ipswitch, MA), and 50 ng of genomic DNA. The PCR cycling program consisted of an initial denaturation at 94 °C for 5 min followed by 30 cycles of 45 s at 94 °C, 45 s at annealing temperature, and 1 min at 72 °C, with a final extension cycle of 72 °C for 5 min. All PCR products were analyzed by electrophoresis in 2 % agarose gels in 1× TAE buffer. Gels were visualized by staining in 0.5 mg/l ethidium bromide and photographed on a digital gel documentation system. The DNA fragment of the expected PCR band was cloned with a pGEM-T kit (Promega, Madison, WI, USA), and the ligation product was transformed into competent cells (Life Technologies, Valencia, CA, USA) followed by culturing overnight at 37 °C. Positive clones containing the expected DNA fragment of the *S*-locus gene were identified by PCR analysis with M13-specific primers and were sequenced at the Plant Biotechnology Institute, National Research Council, Canada. Three to five independent positive clones containing the expected *S*-locus gene DNA fragment from each of the four yellow mustard lines were sequenced to ensure that the correct sequence was obtained. DNA sequence analysis was performed using the BLAST search tool (Blastn, NCBI).

## Results

### Classification of the *S* haplotypes of Y514, Y1130, Y1499, and Y1501

The class I *SLG*- and SRK-specific primer pairs PS5 + PS15 (No. 1, Table 1) and PK1 + PK4 (No. 2, Table 1) amplified a 1.4- and 0.9-kb fragment, respectively, in the SI line Y514; these were designated *SalSLG*-*Y514* and *SalSRK*-*Y514*, respectively. However, these two primer pairs did not generate any PCR amplification in the SI line Y1130 and the SC lines Y1499 and Y1501. The class II *SLG*-specific primer pair PS3 + PS21 (No. 3, Table 1) produced a 1.0-kb fragment in Y1130, Y1499, and Y1501; this fragment was designated *SalSLG*-*Y1130*, *SalSLG*-*Y1499*, and *SalSLG*-*Y1501*, respectively. However, this primer pair failed to generate any band in Y514. Based on these PCR amplification results, we classified the *S* haplotype of Y514 into class I and that of Y1130, Y1499, and Y1501 into class II. To further classify the class II *S* haplotypes of Y1130, Y1499 and Y1501, we used the seven primer pairs (Nos. 4–11, Table 1) to clone the *SRK* gene fragments of the three lines. SRKII-2 produced two bands of 1 and 1.2 kb in both Y1499 and Y1501; these were designated *SalSRK*-*Y1499a*/*b* and *SalSRK*-*Y1501a*/*b*, respectively. SRKII-1, SRKII-6, and SRKII-7 each generated one band of the same size, while PK7-2II + PK8-2II, SRKII-3, SRKII-4, and SRKII-5 showed no amplification in Y1130, Y1499, and Y1501. The class II *SP11* gene-specific primer pair SP11II (No. 12, Table 1) produced one band of 420 bp in Y1130, designated *SalSP11*-*Y1130*, but showed no amplification in Y1499 and Y1501. Based on the PCR amplification results of the class II *SRK* and *SP11* gene-specific primers, we inferred that the *S* haplotype of Y1130 differed from that of Y1499 and Y1501. Cloning and sequencing of *SalSLG*-*Y514*, *SalSRK*-*Y514*, *SalSLG*-*Y1130*, *SalSLG*-*Y1499*, *SalSLG*-*1501*, *SalSP11*-*Y1130*, *SalSRK*-*Y1499a*/*b*, and *SalSRK*-*Y1501a*/*b* confirmed the classification of the *S* haplotypes and provided insight into the divergence of the SC and SI alleles. *SalSLG*-*Y514* was a 1,336-bp fragment which had 70 % similarity with the class II *SalSLG*-*Y1130*, *SalSLG*-*Y1499* and *SalSLG*-*Y1501*. *SalSRK*-*Y514* was a 877-bp fragment. *SalSLG*-*Y1130* consisted of 1,020 bp and shared a similar sequence with *SalSLG*-*Y1499* and *SalSLG*-*Y1501*, but the latter two fragments consisted of only 1,008 bp due to a 12-bp deletion at position 639. It remains to be investigated whether the SC phenotype of Y1499 and Y1501 resulted from the loss-of-function mutation due to the 12-bp deletion of the *SLG* gene. *SalSRK*-*Y1499a*/*b* and *SalSRK*-*Y1501a*/*b* shared the same DNA sequence. *SalSP11*-*Y1130* consisted of 420 bp. Y1499 and Y1501 could have the same *S* haplotype since they were SC and showed no difference in the DNA sequences of the cloned *SLG* and *SRK* gene fragments. Therefore, we inferred that the four yellow mustard inbred lines might represent three different *S* haplotypes: (1) Y514 carried a class I SI *S* haplotype, (2) Y1130 had a class II SI *S* haplotype, (3) Y1499 and Y1501 harbored a class II SC *S* haplotype; these were designated *Sal*-*S1*, *Sal*-*S2* and *Sal*-*S3*, respectively. The *Sal*-*S1SLG*, *Sal*-*S2SLG*, *Sal*-*S3SLG*, *Sal*-*S1SRK*, *Sal*-*S3SRKa*, *Sal*-*S3SRKb*, and *Sal*-*S2SP11* sequences have been deposited in Genbank under accession codes KF355942, KF355943, KF355944, KF355945, KF355946, KF355947, KF355948, respectively.

#### Inheritance of the SI/SC trait

**Fig. 1:**
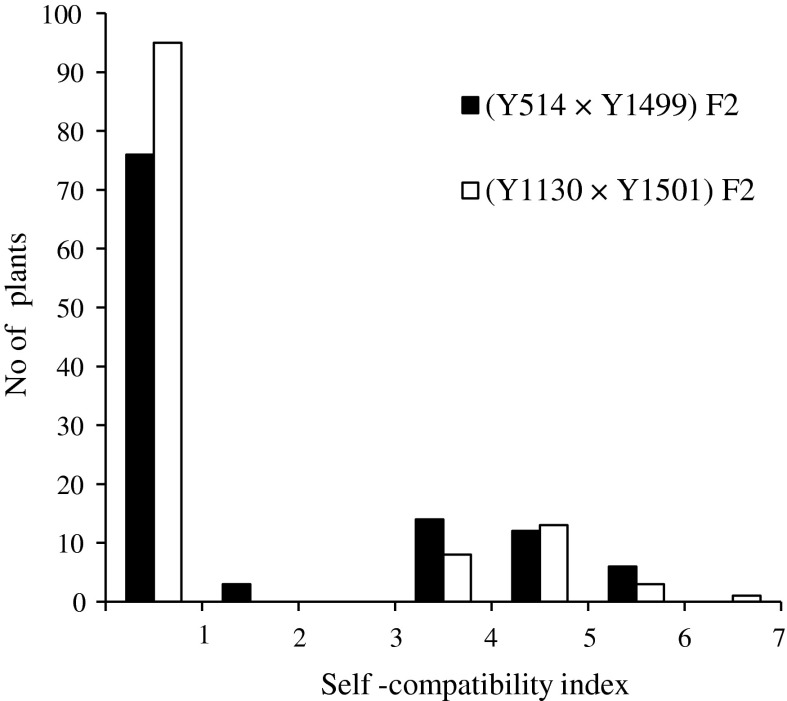
Frequency distribution of self-compatibility index in the F_2_ populations from the crosses of yellow mustard lines Y514 × Y1499 and Y1130 × Y1501

Inheritance of the SI/SC trait was studied in the two crosses of Y514 × Y1499 and Y1130 × Y1501. The SI lines Y514 and Y1130 had a low average SCI of 0.5 and 0.05, respectively. In contrast, the SC lines Y1499 and Y1501 had a high average SCI of 4.2 (range 3.0–6.0) and 4.8 (range 3.2–6.9), respectively. The F_1_ plants of Y514 × Y1499 were SI with an average SCI of 0.5 (range 0.0–1.2), implying the SI *S* haplotype of Y514 was dominant over the SC *S* haplotype of Y1499. The F_2_ plants were classified into two groups: (1) plants with a low SCI (0.0–1.2) similar to that of the SI parent Y514 and (2) plants with a high SCI (3.0–5.9) similar to that of the SC parent Y1499. The ratio of the two groups fit a single-locus gene segregation ratio of 3 SI:1 SC (χ^2^ = 0.87, *P* = 0.35) (Fig. 1). The F_1_ plants derived from the cross Y1130 × Y1501 were also SI with an average SCI of 0.05 (range 0.0–0.1), suggesting the dominance of SI over SC trait in this cross. In the F_2_ generation, plants were divided into two groups: (1) plants with a low SCI (0.0–0.3) similar to that of the SI parent Y1130 and (2) plants with a high SCI (3.2–6.4) similar to that of the SC parent Y1501. The two groups also fit well with the monogenic segregation ratio of 3 SI:1 SC (χ^2^ = 1.11, *P* = 0.29). These inheritance results indicated that the SI/SC trait was controlled by a one-gene locus with SI dominant over SC in the yellow mustard lines studied.

#### Development and validation of the allele-specific markers for the SI/SC phenotype

**Fig. 2:**
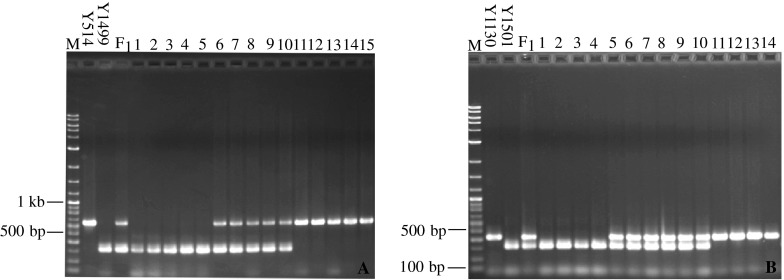
Segregation of co-dominant markers and self-(in)compatibility phenotype in the F_2_ populations of Y514 × Y1499 (**a**) and Y1130 × Y1501 (**b**). **a** Mixing primer pairs Sal-SRKI and Sal-SLGII in a PCR mixture generated co-dominant markers specific for Y514 and Y1499, respectively. *Lanes* *M* DNA ladder, *Y514* SI parent, *Y1499* SC parent, *F*_*1*_ *plant* Y514 × Y1499, *1*–*5* homozygote SC F_2_ plants with a marker specific for the SC parent Y1499, *6*–*10* heterozygote SI F_2_ plants with markers specific for both parents, *11*–*15* homozygote SI F_2_ plants with markers specific for the SI parent Y514. **b** Mixing primer pairs Sal-SLGII and Sal-SP11II in a multiple PCR mixture generated markers specific for Y1130 and Y1501, respectively. *Lanes* *M* DNA ladder, *Y1130* SI parent, *Y1501* SC parent, *F*_*1*_ *plant* Y1130 × Y1501, *1*–*4* homozygote SC F_2_ plants with a marker specific for the SC parent Y1501, *5*–*10* heterozygote SI F_2_ plants with markers for Y1130 and Y1501, respectively, *11*–*14* homozygote of SI F_2_ plants with a marker specific for the SI parent Y1130

*Allele*-*specific markers for the SI phenotype of Y514 and Y1130* Based on the sequence information of *SalSLG*-*Y514* and *SalSRK*-*Y514*, primer pairs Sal-SLGI and Sal-SRKI (Table 2) were designed to generate diagnostic markers specific for the SI phenotype of Y514. The two primers each produced one dominant marker of 626 and 640 bp, respectively, in Y514, but did not generate any amplification in Y1130, Y1499, and Y1501 (Table 3). Co-segregation of the two markers and the SI phenotype was studied in the F_2_ population derived from the cross of Y514 × Y1499 (Fig. 2a). As expected, the SI allele-specific marker was present in the F_1_ and all SI F_2_ plants, but absent in all SC F_2_ plants. The dominant marker generated by the primer pair Sal-SRKI was linked to the SI phenotype of Y514 in the cross Y514 × Y1499 [Electronic Supplementary Material (ESM) Fig. S1A]. The primer pair Sal-SP11II generated one dominant marker of 420 bp that was specific for Y1130 (Table 3). Co-segregation of this marker and the SI phenotype was confirmed in the F_2_ population of Y1130 × Y1501. The dominant marker was present in the F_1_ and all SI F_2_ plants, but absent in all SC F_2_ plants (ESM Fig. S1B).

**Table 3:** The SC/SI phenotype, *S* haplotype, and allele-specific markers of the four parental lines

Line	SI/SC phenotype	*S* haplotype	Allele-specific markers for SI/SC phenotype				
			Sal-SLGI	Sal-SRKI	Sal-SLGII	Sal-SRKII	Sal-SP11II
Y514	SI	Class I, *Sal*-*S1*	+	+	−	−	−
Y1130	SI	Class I, *Sal*-*S2*	−	−	−	−	+
Y1499	SC	Class II, *Sal*-*S3*	−	−	+	+	−
Y1501	SC	Class II, *Sal*-*S3*	−	−	+	+	−

*SC* self-compatible, *SI* self-incompatible, + present, − absent

*Allele*-*specific markers for the SC phenotype of Y1499 and Y1501* The 12-bp deletion in the *SLG* gene fragment of Y1499 and Y1501 was used to design the SC allele-specific markers. As expected, the primer pair Sal-SLGII spanning the 12-bp deletion region generated a 310-bp fragment specific for the SC lines Y1499 and Y1501 (Table 3). Sal-SRKII revealed two polymorphic bands in Y1499 and Y1501 (Table 3). To validate the SC allele-specific markers generated by the two primer pairs Sal-SLGII and Sal-SRKII, we studied co-segregation of these markers with the SC phenotype in the two crosses of Y514 × Y1499 and Y1130 × Y1501. As indicated in ESM Fig. S1C, F_1_ plants and all SC F_2_ plants exhibited the SC allele-specific marker produced by primer pair Sal-SLGII. The homozygote SI F_2_ plants did not show any amplification. However, due to the dominance of SI over SC, the heterozygote SI F_2_ plants had the SC allele-specific marker and could not be distinguished from the homozygote SC genotype at the seedling stage. These results indicated that the SC allele-specific dominant markers could be useful for marker-assisted selection of SC lines in yellow mustard breeding. However, co-dominant markers need to be developed in order to distinguish the homozygote SC from heterozygote SC genotypes.

#### Development of co-dominant markers using multiplex PCR

The development of *S*-locus gene-based co-dominant markers is essential for the determination of zygosity, which is needed in breeding programs. Multiplex PCR has proven to be a very useful molecular tool for the amplification of multiple targets in a single PCR experiment. In a multiplexing assay, more than one target sequence can be amplified through the use of multiple polymorphic primers present in the reaction mixture. In our study, we successfully developed four co-dominant markers using the multiplex PCR method. Sal-SRKI generated one dominant marker for the SI parent Y514, while Sal-SLGII and Sal-SRKII produced dominant marker(s) specific for the SC parents Y1499 and Y1501 (Table 3). These results indicate that including Sal-SRKI and Sal-SLGII or Sal-SRKI and Sal-SRKII in the PCR reaction would result in the production of co-dominant markers specific for each parent in the F_1_ plant of Y514 × Y1499. The co-dominant markers generated by mixing Sal-SRKI and Sal-SLGII in the PCR reaction were then used for zygosity determination in the F_2_ population derived from Y514 × Y1499 (Fig. 2a). Based on this analysis, we classified these F_2_ plants into three types: (1) 22 homozygote SI plants which exhibited the dominant 640-bp marker specific for Y514; (2) 57 heterozygote SI plants which showed the two markers, 640 and 310 bp, specific for Y514 and Y1499, respectively; (3) 32 homozygote SC plants which had the dominant 310-bp marker specific for line Y1499. Segregation of the three genotypes fit well with the ratio of 1:2:1 (χ^2^ = 1.88, *P* = 0.39). Sal-SP11II produced one dominant marker of 420 bp that was specific for Y1130. These results indicate that the PCR mixture containing Sal-SLGII and Sal-SP11II or Sal-SRKII and Sal-SP11II was able to produce co-dominant markers specific for both parents in the F_1_ plants of Y1130 × Y1501. The co-dominant markers generated by mixing Sal-SLGII and Sal-SP11II were used to determine the zygosity of F_2_ plants derived from the cross Y1130 × Y1501 (Fig. 2b). Based on this analysis, we classified these F_2_ plants into three groups: (1) 27 homozygote SI plants which showed the dominant marker (420 bp) of the SI parent Y1130, (2) 68 heterozygote SI plants which had markers specific for both Y1130 and Y1501, (3) 25 homozygote SC plants which had the dominant marker (310 bp) of Y1501. The segregation ratio of the three groups fit well with the segregation ratio of 1:2:1 (χ^2^ = 2.2, *P* = 0.33).

## Discussion

Genetically stable SI and SC inbred lines have been developed via pedigree breeding in yellow mustard (Cheng et al. 2012). In the present study, we cloned the *SLG*, *SRK*, and *SP11* gene fragments and then sequenced these in the four yellow mustard lines Y514, Y1130, Y1499, and Y1501 using the class I and II *S*-locus gene-specific primers from *B. rapa* and *B. oleracea*. Based on the PCR amplification and sequencing results, Y514 could be classified as a class I *S* haplotype whereas Y1130, Y1499, and Y1501 were classified as a class II *S* haplotype. The deduced amino acid sequence of the *SLG* gene of Y514 had only about a 60 % similarity with those of Y1130, Y1499, and Y1501 (ESM Fig. S2), suggesting the occurrence of sequence divergence between class I and II *S* haplotypes in yellow mustard, which is in agreement with earlier reported findings in *B. rapa* and *B. oleracea* (Nasrallah et al. 1991; Nasrallah and Nasrallah 1993). Sequence comparisons of the *SLG*, *SRK*, and *SP11* genes between yellow mustard and *Brassica* species identified the corresponding *S* haplotypes. The *SLG* (GenBank accession no. KF355942) and *SRK* (GenBank accession no. KF355945) gene sequences of Y514 were 92 and 95 % identical with the *SLG* gene (GenBank accession no. AB054734) and *SRK* gene (GenBank accession no. JX416335) of the class I *S* haplotype *S*-*57* in *B. oleracea*, implying that the *S* haplotype of Y514 and *S*-*57* in *B. oleracea* could be phylogenetically related. Y1130, Y1499, and Y1501 were of the class II *S* haplotype. The *S*-locus gene sequences of these three lines were compared with those of class II *S* haplotypes in *B. rapa*. The *SLG* gene sequences (GenBank accession no. KF355943 and GenBank accession no. KF355944) of the three lines showed 92 % identity to *BrSLG*-*60* (GenBank accession no. AB097116; Fukai et al. 2003). The *SRK* sequences (*Sal*-*S3SRKa* and *Sal*-*S3SRKb*; GenBank accession no. KF355946 and GenBank accession no. KF355947) of Y1499 and Y1501 exhibited 89 % similarity to *BrSRK60* (GenBank accession no. AB097116; Fukai et al. 2003), and the *SP11* gene (GenBank accession no. KF355948) of Y1130 showed 93 % identity with *BrSP11*-*60* (GenBank accession no. AB067446; Shiba et al. 2002). These results suggested that the *S* haplotype of Y1130, Y1499, and Y1501 could correspond to class II *S* haplotype *S*-*60* in *B. rapa*. Compared with the class II *S* haplotypes in *B. oleracea*, the *SRK* gene of Y1499 and Y1501 had 89 % identity to *BoSRK*-*15* (GenBank accession no. AB180903; Fujimoto et al. 2006), and the *SP11* gene of Y1130 showed 94 % similarity to *BoSP11*-*15A* (GenBank accession no. AB180904; Fujimoto et al. 2006). As expected, the deduced amino acid sequences of the *S*-locus genes of Y514, Y1130, Y1499, and Y1501 had a high similarity with those of their corresponding *S* haplotypes *S*-*57*, *S*-*60*, and *S*-*15* in *Brassica* species (ESM Fig. S2). This implied that the *S* haplotype of Y1130, Y1499, and Y1501 could be related to the class II *S* haplotype *S*-*15* in *B. oleracea*. Sato et al. (2003) reported that *S*-*60* in *B. rapa* and *S*-*15* in *B. oleracea* are phylogenetically related. Therefore, it is possible that the *S* haplotype of Y1130, Y1499, and Y1501 in yellow mustard, *S*-*60* in *B. rapa*, and *S*-*15* in *B. oleracea* have the same evolutionary origin. Our study provides additional evidence that yellow mustard is phylogenetically related to *Brassica* species (Warwick and Black 1991). However, the primer pairs PK7-2II + PK8-2II, SRKII-3, SRKII-4, and SRKII-5 (Table 1), which were designed to amplify the *SRK* gene in *B. rapa* and *B. oleracea*, failed to amplify the expected fragments in yellow mustard, suggesting the occurrence of divergence of the *S* haplotypes after the speciation of yellow mustard.

The inheritance of the SI/SC trait was studied in the two crosses of Y514 × Y1499 and Y1130 × Y1501. The class II SC *S* haplotype of Y1499 and Y1501 was found to be recessive to the class I and II SI *S* haplotypes of Y514 and Y1130, respectively, and to be controlled by a one-gene locus, suggesting that the inheritance relationship between the class I and II *S* haplotypes in yellow mustard is consistent with that reported in *Brassica* species (Bateman 1955; Nasrallah and Nasrallah 1993). This knowledge is of great relevance for yellow mustard breeding. The SC gene sources controlled by a single gene could be transferred into elite SI lines to develop SC varieties with improved agronomic and quality traits in yellow mustard since desirable traits can be more easily fixed in SC lines than in SI ones. This will allow the approach of commercial yellow mustard breeding programs to be switched from improving outcrossing populations to developing more uniform elite inbred line varieties. In addition, the availability of dominant and recessive SC gene sources in *B. napus* would allow the self-incompatibility reproduction system to be used to produce three-way hybrids (Fu 1981; Zhang et al. 2011). In such a system, the recessive SC gene source is used as maintainer (B) for the SI line while the dominant SC gene is used as restorer (R) line to produce SC F_1_ hybrids. In yellow mustard, the recessive SC gene sources of Y1499 and Y1501 could be used to develop maintainers (B) for the SI lines once a dominant SC gene source has been identified and could also be used as restorer (R) line; this would allow the development of three-way hybrids.

Lines Y1499 and Y1501 had a recessive SC phenotype. Two primer pairs, Sal-SLGII and Sal-SRKII, were designed based on the DNA sequence polymorphism of the *SLG* and *SRK* genes and used to generate dominant markers specific for the SC phenotype of Y1499 and Y1501. We have demonstrated that the dominant markers co-segregated with the SC phenotype in the F_2_ populations of Y514 × Y1499 and Y1130 × Y1501. Multiplex PCR containing two polymorphic primers was used to generate co-dominant markers linked to the SI/SC trait in *B. napus* (Zhang et al. 2008). In our studies, we developed co-dominant markers for the SI/SC trait by mixing the two informative primers specific for SI and SC parents in the same PCR reaction mixture. These *S*-locus gene-based co-dominant markers are currently being used for marker assisted selection in our yellow mustard breeding program. In the F_2_ generation, the homozygote SC and heterozygote SI plants of interest can be identified and selected for further breeding, whereas the homozygote SI plants can be discarded at the seedling stage in the greenhouse, thereby greatly enhancing breeding efficiency.

Different inbred lines have been produced and will be used for developing high-yielding synthetic varieties in yellow mustard. Characterization of the *S* haplotypes of different inbred lines is essential for the selection of synthetic component lines. The *S* locus gene-specific primers developed in this study will be used to characterize the *S* haplotypes of various elite inbred lines in yellow mustard.

## Electronic supplementary material

Below is the link to the electronic supplementary material.

Supplementary material 1 (DOC 471 kb)
