# Supplementary material for: Self-(in)compatibility inheritance and allele-specific marker development in yellow mustard (Sinapis alba)
Source: Mol Breed. 2013 Sep 22;33(1):187–96. doi: 10.1007/s11032-013-9943-8 (PMC3890562; doi:10.1007/s11032-013-9943-8)
Supplement: Supplementary file 1 — Supplementary material 1 (DOC 471 kb) [file 11032_2013_9943_MOESM1_ESM.doc]

**Self-(in)compatibility inheritance and allele-specific marker development in yellow mustard (*Sinapis alba*)**

**Molecular Breeding**

Fangqin Zeng and Bifang Cheng*

Agriculture and Agri-Food Canada, Saskatoon Research Centre, 107 Science Place, Saskatoon, SK, S7N 0X2, Canada

*Corresponding author: Research Scientist, Saskatoon Research Centre, Agriculture and Agri-Food Canada, 107 Science Place, Saskatoon, Saskatchewan, Canada, S7N 0X2

Telephone: 306-956-7691

Facsimile: 306-956-7247

E-mail address: bifang.cheng@agr.gc.ca


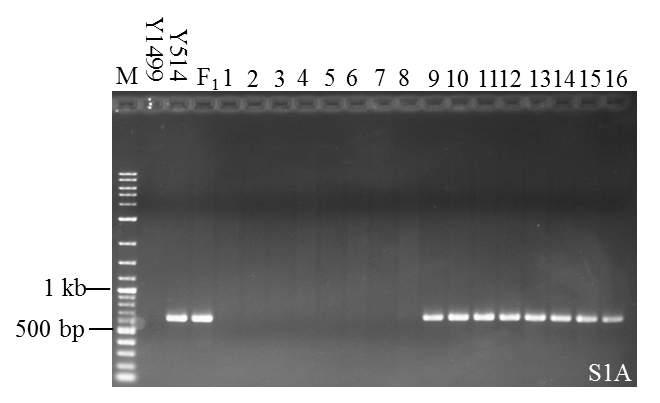


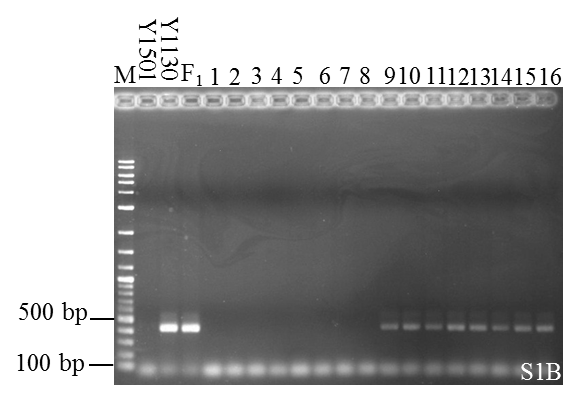


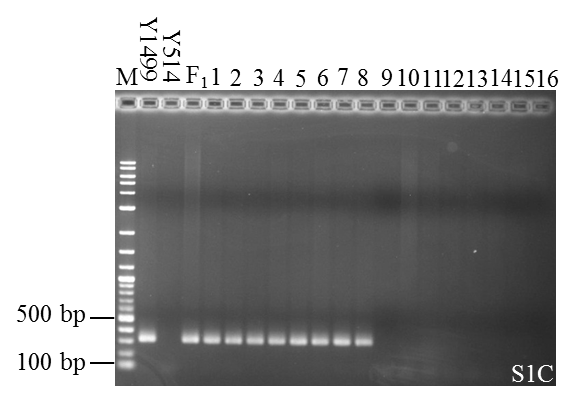


**EMS Fig. S1** Co-segregation of *S*-locus gene based markers and SI/SC phenotype in the F_2_ populations of Y514 ×Y1499 and Y1130 × Y1501

1A: The dominant marker generated by the primer pair Sal-SRKI was linked to the SI phenotype of Y514 in the cross Y514 ×Y1499. M: DNA ladder; Y1499: SC parent; Y514: SI parent; F_1_ plant: Y514 × Y1499; Lanes 1-8: SC F_2_ plants; Lanes 9-16: SI F_2_ plants.

1B: The dominant marker generated by the primer pair Sal-SP11II was linked to the SI phenotype of line Y1130 in the cross Y1130 × Y1501. M: DNA ladder; Y1501: SC parent; Y1130: SI parent; F_1_ plant: Y1130 × Y1501; Lanes 1-8: SC F_2_ plants; Lanes 9-16: SI F_2_ plants.

1C: The dominant marker generated by the primer pair Sal-SLGII was linked to the SC phenotype of line Y1499 in the cross Y514 × Y1499. M: DNA ladder; Y1499: SC parent; Y514: SI parent; F_1_: Y514 × Y1499; Lanes 1-8: SC F_2_ plants; Lanes 9-16: SI F_2_ plants.


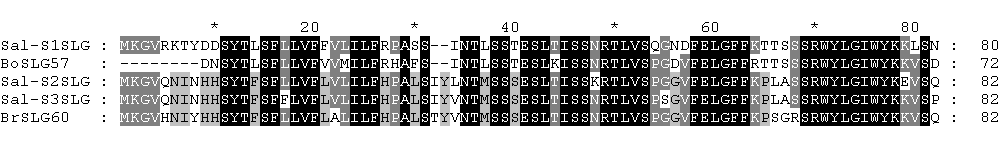


A


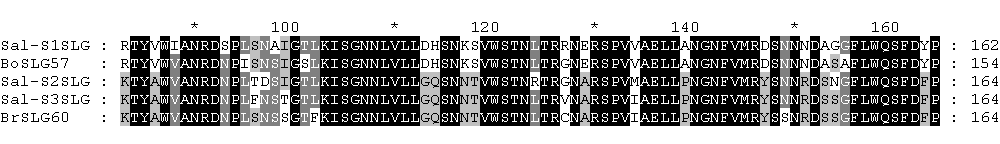


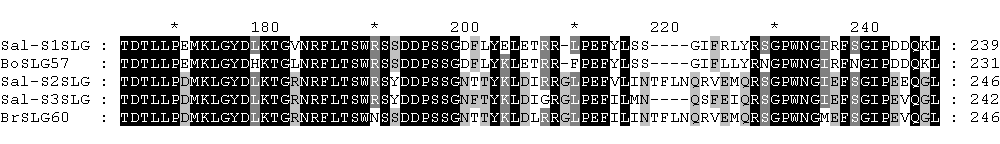


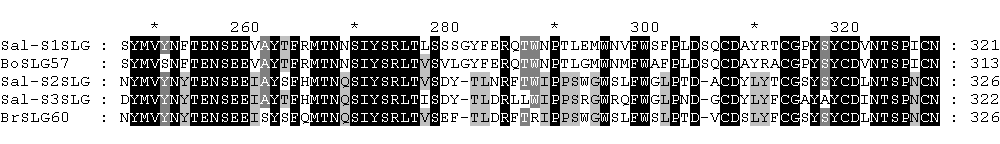


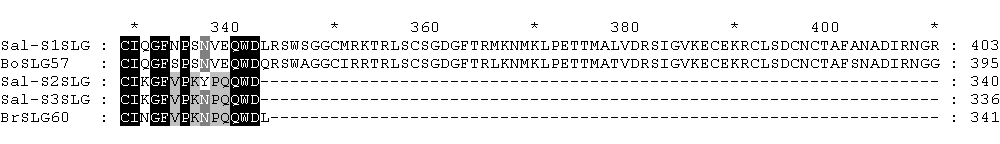


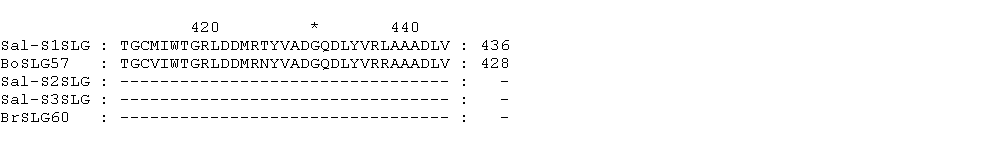


B


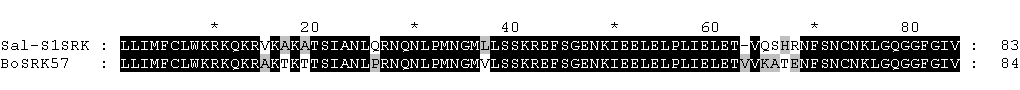


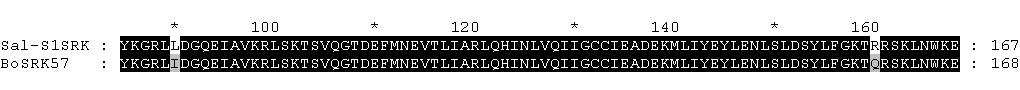


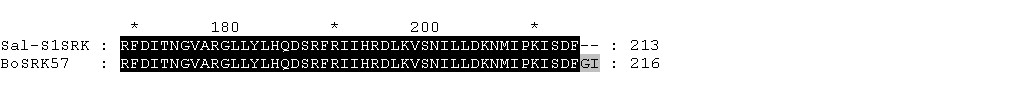


C


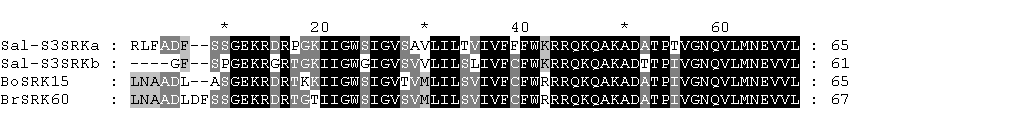


D


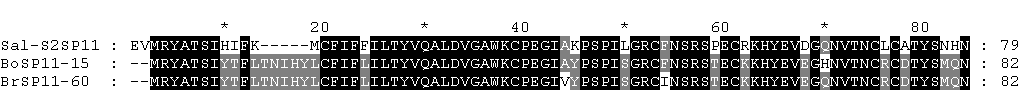


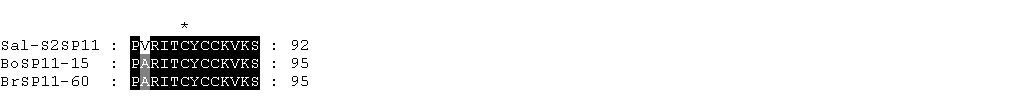


**EMS Fig. S2** Alignment of the deduced amino acid sequences of *S*-locus genes in *S. alba* and *Brassica* species.

2A: Alignment of the deduced amino acid sequences of *SLG* genes from *S. alba* *Sal-S1*, *Sal-S2*, *Sal-S3* (Table 3), *B. oleracea S-57* and *B. rapa S-60*.

2B: Alignment of the deduced amino acid sequences of *SRK* genes from *S. alba* *Sal-S1* (Table 3) and *B. oleracea S-57*.

2C: Alignment of the deduced amino acid sequences of *SRK* genes from *S. alba* *Sal-S3* (Table 3), *B. oleracea* *S-15* and *B. rapa S-60*.

2D: Alignment of the deduced amino acid sequences of *SP11* genes from *S. alba* *Sal-S2* (Table 3), *B. oleracea* *S-15* and *B. rapa S-60*.
